# Effects of high fat diet-induced obesity on vitamin D metabolism and tissue distribution in vitamin D deficient or supplemented mice

**DOI:** 10.1186/s12986-020-00463-x

**Published:** 2020-06-15

**Authors:** Chan Yoon Park, Yongho Shin, Jeong-Han Kim, Shuang Zhu, Young Sun Jung, Sung Nim Han

**Affiliations:** 1 Department of Food and Nutrition, College of Human Ecology, Seoul National University, 1 Gwanak-ro, Gwanak-gu, Seoul, South Korea; 2 Department of Agricultural Biotechnology, College of Agriculture and Life Science, Seoul National University, Seoul, South Korea; 3 Research Institute of Human Ecology, Seoul National University, Seoul, South Korea

**Keywords:** Vitamin D supplementation, Obesity, Tissue vitamin D, 25-hydroxyvitamin D, Vitamin D absorption

## Abstract

**Background:**

Vitamin D deficiency has been often observed in obese persons. One of the mechanisms suggested for low vitamin D status in obesity was decreased bioavailability of vitamin D (VD) due to sequestration in adipose tissue. However, only few studies have investigated this mechanism via quantifying vitamin D levels from tissues from the obese.

**Methods:**

Six-wk-old C57BL/6 mice were fed 10 or 45% kcal fat (CON or HFD) diets containing 50, 1000 or 25,000 IU vitamin D3/kg diet (LVd, CVd or HVd) for 13 wks. Serum 25-hydroxyvitamin D (25(OH)D) levels were determined by radioimmunoassay and liver and adipose tissue cholecalciferol (VD3) and 25-hydrocholecalciferol (25(OH)D3) levels were measured by LC-MS/MS. mRNA levels of jejunal *Mttp, Cd36, Sr-b1, Npc1l1*, and *Abca1* and liver and adipose tissue 25-hydroxylases (*Cyp2r1* and *Cyp27a1*) were determined by real-time PCR.

**Results:**

Serum 25(OH)D levels were affected by dietary vitamin D content but differential effects were observed between HFD and CON groups. When vitamin D intake was at a supplementary level, the HFD-HVd group had lower serum 25(OH)D levels than the CON-HVd group, while there was no significant difference between the HFD and CON groups fed LVd or CVd. Total amount of VD3 in liver and adipose tissue were significantly higher in HFD-HVd group compared with the CON-HVd group. However, no difference in total amount of tissue VD3 was observed between the CON and HFD groups fed CVd. In jejunum, mRNA levels of *Mttp* and *Abca1* were significantly higher in HFD groups than CON groups. There was no difference in mRNA levels of liver 25-hydroxylases by both dietary fat amount and vitamin D content.

**Conclusion:**

A significant amount of VD3 seemed to be stored in the liver and adipose tissue when dietary vitamin D is at a supplementation level; thus excess body adiposity could contribute to relatively low serum 25(OH)D level when vitamin D was supplemented.

## Background

Vitamin D (VD) insufficiency (serum 25-hydroxyvitamin D (25(OH)D) levels lower than 30 ng/mL) is highly prevalent worldwide and it has been estimated that more than 30–50% of the populations in Europe and the US, and more than 50% in East Asia are VD insufficient or deficient [1–3]. Low serum 25(OH)D levels have been observed in obese persons and circulating 25(OH)D levels correlated inversely with body mass index and body fat mass [4–6]. It has been reported that the prevalence of VD deficiency was 35% higher in obese persons and 24% higher in overweight persons compared with non-obese persons [7]. Although several mechanisms such as low ultraviolet B (UVB) exposure of obese people and VD sequestration or dilution into adipose tissue have been proposed for the higher prevalence of VD deficiency among the obese, the causes of their low 25(OH)D levels are still inconclusive.

Early studies on adipose tissue using animals or humans supplemented with ^14^C-radiolabeled cholecalciferol (VD3) followed by measuring the radioactivity have shown that VD and its metabolites are stored in adipose tissue [8, 9]. Wortsman et al. [10] observed that serum 25(OH)D levels increased more in a non-obese group compared with an obese group when they were on exposure to UVB or when VD supplements were given to them. Thus, it has been suggested that adiposity could lead to the decreased bioavailability of VD due to VD sequestration in body fat. Furthermore, Drincic et al. [6] proposed volumetric dilution (VD distributed over a larger body size) as the reason for low serum 25(OH)D concentration in the obese. An inverse association between serum 25(OH)D levels and body fat mass using the hyperbolic fit model (the mathematical expression of dilution) was used for explaining low VD status associated with obesity. Although VD sequestration or volumetric dilution might explain the VD deficiency observed in the obese, few studies have directly demonstrated these hypotheses by quantifying VD levels in adipose tissue and comparing VD levels between obese and non-obese subjects due to the difficulty in measuring fat-soluble VD from small quantities of fat tissue [11–13]. In human studies, it is difficult to control for the variables related to VD synthesis in the skin, such as UVB exposure time, usage of sunblock, season, and age. Therefore, environmentally controllable animal studies are needed to provide evidence for how obesity affects the storage of VD in soft tissues and VD metabolism.

VD is fat-soluble and is usually absorbed with dietary fat by passive diffusion in proximal jejunum and distal ileum [14]. Dietary VD is packaged into chylomicrons with triglycerides, cholesterol, and other lipids within the intestine wall, and this process is dependent on microsomal triglyceride transfer protein (MTP) [15–17]. VD contained in chylomicron is secreted into lymphatic system and some of them are taken into adipose tissue and liver during chylomicron hydrolysis by lipoprotein lipase (LPL). Fat intake could influence the absorption of dietary VD in the small intestine and the impact of the fat amount in a meal on serum VD status has been investigated in several studies, although the results remain inconclusive. Raimundo et al. [18] reported that a single oral intake of 50,000 IU VD3 with a high-fat meal containing 25.6 g fat/meal resulted in a significant increase in serum 25(OH)D levels after 14 days, while there was no significant change in 25(OH)D levels when VD was taken with a low-fat meal (1.7 g fat/meal). Dawson-Hughes et al. [19] observed that plasma VD3 level at 12 h after taking 50,000 IU VD3 tablet with a low-fat meal (10% kcal, 11.1 g total fat) was 20% higher when compared with the plasma VD3 levels after VD3 taken with no meal or a high-fat meal (50% kcal, 35.2 g total fat). Recent studies have suggested the possibility of VD absorption through protein-mediated diffusion by cholesterol transporter proteins such as scavenger receptor class B type 1 (SR-B1), cluster of differentiation 36 (CD36), and Niemann-Pick C1-Like 1 (NPC1L1) and their expression levels could be affected by obesity or the amount of fat intake [20, 21]. Therefore, in addition to adiposity, dietary fat amount and the expression of transporter proteins involved in VD absorption should be considered for the circulatory 25(OH)D response to VD intake.

The objective of this study was to investigate whether obesity affects VD metabolism and tissue distribution when dietary VD was at deficient or supplementation levels. To achieve this purpose, circulatory and tissue levels of vitamin D and expression of genes involved in vitamin D absorption and metabolism were determined in obese and control mice fed different levels of vitamin D.

## Methods

### Animals and diets

Six-week-old C57BL/6 male mice (Central Animal Laboratory, Seoul, Korea) were housed in the specific-pathogen-free animal facility at Seoul National University with environmentally controlled temperature (23 ± 1 °C), relative humidity (50 ± 10%), and a 12-h light/12-h dark cycle. After 5 d of acclimation with the control diet, the mice were assigned to 6 groups and fed control (10% kcal fat, CON) or high-fat (45% kcal fat, HFD) diets containing three different levels of VD3 [low (50 IU/kg diet, LVd), control (1000 IU/kg diet, CVd), or high (25,000 IU/kg diet, HVd)] ad libitum for 13 wk. (CON-LVd, #119320; CON-CVd, #103816; CON-HVd, #119321; HFD-LVd, #119318; HFD-CVd, #103818; HFD-HVd, #119319; Dyets, Inc., Bethlehem, PA, USA) (See additional file 1: Table S1). Food intake was measured 4 times per week and body weight was recorded weekly. Food intake in mice was measured by providing known amount of food and subtracting the weight of remaining food. At the end of the 13th week, the animals were fasted for 12 h and two animals from each group were anesthetized with 1.5 g/ kg urethane (intraperitoneal), then scanned with a Hologic Discovery DEXA instrument (Hologic, Toronto, ON, Canada). After 12 h of fasting, all mice were euthanized by CO_2_ asphyxiation. Blood, liver, small intestine (duodenum, jejunum, and ileum), kidney, and adipose tissue were collected, weighed, and stored at − 80 °C until analyzed.

### Determination of liver triacylglycerol (TG) levels

Lipid was extracted from the liver according to the method by Folch et al. [22], then liver TG levels were determined using a commercial kit (Asan pharmaceutical, Seoul, Korea) based on an enzymatic assay.

### Determination of serum 25(OH)D levels

Serum 25(OH)D levels were measured via a radioimmunoassay (RIA) using commercial RIA kit (DiaSorin, Stillwater, MN, USA) according to the manufacturer’s instructions. The radioactivity was measured with an automatic gamma counter (2470 Wizard2, Perkin Elmer, Shelton, CT, USA).

### Determination of vitamin D**3** and 25(OH)D**3** levels in liver and adipose tissue

#### Chemicals and reagents

VD3, 25(OH)D3, isotopically labeled 6, 19, 19 [d3]-VD3, d3–25(OH)D3, methyl tert-butyl ether (MTBE), formic acid (LC-MS grade), and ammonium formate (≥99.0%) were purchased from Sigma-Aldrich (St. Louis, MO, USA). Methanol, ethanol, and acetonitrile (AcN) were liquid chromatography/mass spectrometry (LC/MS)-grade and purchased from Fisher Scientific (Pittsburgh, PA, USA). Ultra-pure water was prepared with LaboStar™ TWF UV 7 (Siemens, MA, USA).

#### Sample preparation and PTAD derivatization

VD3 and 25(OH)D3 were analyzed from liver and adipose tissue using a modification of the original method by Lipkie et al. [12]. Quality control (QC) samples used for the external standard curve were prepared from the adipose tissue and liver of 22-week-old C57BL6 mice fed a VD-deficient diet (50 IU VD3/kg diet). Detailed procedure is described in additional file [Additional file 1- Method].

#### LC-MS/MS analysis

A Shimadzu LCMS-8040 triple quadrupole mass spectrometer with a Shimadzu Nexara X ultra-high-performance liquid chromatography (UHPLC; Kyoto, Japan) system was used for the separation of PTAD-derivatized VD3 and 25(OH)D3. In the UHPLC system, a Kinetex® C18 column (100 × 2.1 mm, 2.6 μm, Phenomenex, Torrance, CA, USA) with a SecurityGuard™ Ultra guard column (Phenomenex) was used to separate each target analyte peak. The oven temperature was 40 °C. Mobile phase A was 0.1% formic acid and 5 mM methylamine in water and B was 0.1% formic acid in methanol, and the flow rate was 0.2 mL/min. The gradient for B was started with 20% total volume and 30 s later, %B was raised to 70% for 0.5 min, ramped to 99% for 17.5 min, and held for 3 min. Next, %B was dropped sharply to 20% for 0.5 min and held for 5 min. The total run time was 27 min. The injection volume was 5 μL.

In the MS system, electrospray (ESI)-positive mode was used for the ionization of the target compounds. The nebulizing and drying gas (nitrogen) flows were 3 and 15 L/min, respectively. The desolvation line and heat block temperature were 250 and 400 °C, respectively. The collision-induced dissociation gas was argon. Scheduled multiple reaction monitoring (MRM) was used for each analyte, and its detection window and dwell time were ± 1 min and 10 ms, respectively. LCMS software (version 5.60, LabSolutions) was used for data processing.

#### Validation of vitamin D**3** and 25(OH)D**3** quantification method

To validate the accuracy of the method, liver or adipose tissue was homogenized in PBS to 33% (w/w) solutions. 10 μL of internal standard (a mixture of d3-VD3 and d3–25(OH)D3, 1 μg/ mL) was added to 400 μL of homogenate, then spiked with 10 μL of 2, 10, or 100 ng/ mL of d3-VD3 and d3–25(OH)D3 mixture in AcN (final spiked levels: 1.5, 7.5, or 75 ng/g tissue). The endogenous VD3 level was determined from the sample with only the internal standard added. Accuracy was evaluated with 3 samples, repeated over 3 days, by the following equation: (mean of measured value/QC sample value) × 100%.

Within-day precision was determined by analyzing the same liver and epididymal fat homogenates three separate times in 1 day. Between-day precision was determined by analyzing the same QC sample on 3 different days. Precision was determined as the relative coefficient of variation (%CV): (standard deviation/mean) × 100%.

### RNA extraction and real time PCR

Total RNA was extracted using Trizol reagent (Invitrogen, Carlsbad, CA, USA). The quality of the RNA sample was determined using agarose gel electrophoresis with the Gel Doc XR system (Bio-Rad Laboratories, Hercules, CA, USA), and the absorbance at 260 and 280 nm with a nano-spectrophotometer (NANO-200, BIOAND Co., Gyeonggi, Korea). cDNA was synthesized using a PrimeScript™II 1st strand cDNA synthesis kit (Takara Bio., Shiga, Japan) with a thermal cycler (Applied Biosystems, Foster City, CA, USA). mRNA levels of cytochrome P450 2R1 (*Cyp2r1*), cytochrome P450 27A1 (*Cyp27a1*), low density lipoprotein receptor-related protein 1 (*Lrp1*), and VD binding protein (*Dbp*) in the liver, lipoprotein lipase (*Lpl*) in the adipose tissue, microsomal triglyceride transfer protein (*Mttp*), cluster of differentiation 36 (*Cd36*), scavenger receptor class B member 1 (*Sr-b1*), Niemann-Pick C1-Like 1 (*Npc1l1*), and ATP-binding cassette transporter (*Abca1*) in the jejunum were determined using real-time PCR with SYBR Premix Ex Taq (Takara bio.) and StepOne Real-time PCR System (Applied Biosystems). All values were normalized to the levels of *Gapdh* and expressed as relative mRNA level compared to the average level of the CON-CVd group. The sequences of primers used in this study are presented in Additional file 1: Table S2.

### Statistical analyses

All data were analyzed using SPSS statistical software version 23.0 (IBM SPSS Statistics, Chicago, IL, USA). Normality in distribution was determined based on the results of a Shapiro-Wilk test and Q-Q plot. For data not following the normal distribution (*Sr-b1* and *Lpl*), square root transformation was done for statistical analysis. To evaluate the overall effects of dietary fat amount and VD content, and their interaction, comparisons were made by two-way ANOVAs. Further individual comparisons among groups were done by Fisher’s least significant difference post hoc test. For analyses of correlations between serum 25(OH)D level (dependent variable) and other factors, Pearson’s correlation was used to determine the association between parameters. All data are presented as means ± SEMs and *P* <  0.05 was considered as statistically significant.

## Results

### Body weight, adipose tissue and liver weights, food intake, and liver TG levels

There was no significant difference in body weight at week 0 among the groups. The HFD groups had 38.7% higher body weight (*P* <  0.001) and 107.4% higher white adipose tissue weight (*P* <  0.001) compared with the CON groups. Overall, there was no statistically significant effect of dietary VD content on body weight and adipose tissue weight (Table 1), and the average daily food intake (g/day) was not affected by dietary VD content or fat amount. Based on the VD content of the experimental diets and daily food intake (g/day), the actual daily VD intake (IU/day) calculated was 0.15, 2.8, or 71.3 IU/day in the CON groups (LVd, CVd, or HVd, respectively) and 0.18, 3.7, or 82.5 IU/day in the HFD groups (LVd, CVd, or HVd, respectively). Liver weight per body weight (g/100 g body weight) and liver TG levels were significantly higher in the HFD groups than the CON groups. Overall, VD content in the diet did not affect the liver weight or liver TG levels.

**Table 1:** Body weight (wt.), white adipose tissue (WAT) wt., dietary intake, liver wt., and liver TG levels in the CON and HFD groups fed different levels of vitamin D^1^

	CON			HFD			***P***-value		
	LVd (***n*** = 9)	CVd (***n*** = 11)	HVd (***n*** = 8)	LVd (***n*** = 9)	CVd (***n*** = 11)	HVd (***n*** = 8)	Fat amount	Vitamin D content	Interaction
Body wt. at 0 wk. (g)	22.6 ± 0.4	23.1 ± 0.4	23.2 ± 0.2	22.7 ± 0.3	22.6 ± 0.2	22.7 ± 0.7	0.24	0.56	0.49
Body wt. at 13 wk. (g)	35.8 ± 1.0^a^	35.0 ± 0.4^a^	34.5 ± 1.1^a^	49.5 ± 0.7^c^	49.5 ± 0.9^c^	46.7 ± 0.8^b^	< 0.001	0.06	0.42
WAT wt^2^ (g)	2.91 ± 0.31^a^	2.74 ± 0.22^a^	2.64 ± 0.29^a^	5.59 ± 0.09^b^	5.90 ± 0.11^b^	5.70 ± 0.21^b^	< 0.001	0.79	0.50
Daily food intake (g/day)	2.96 ± 0.04	2.92 ± 0.05	2.98 ± 0.04	3.03 ± 0.05	3.11 ± 0.11	2.80 ± 0.05	0.66	0.18	0.036
Liver wt./body wt. (%)	3.23 ± 0.14^a^	3.41 ± 0.14^a^	3.34 ± 0.09^a^	5.24 ± 0.13^c^	4.74 ± 0.23^bc^	4.37 ± 0.29^b^	< 0.001	0.16	0.038
Liver TG levels (mg/g tissue)	57.2 ± 5.0^a^	54.6 ± 8.7^a^	63.6 ± 6.4^a^	138.3 ± 10.1^b^	127.7 ± 12.9^b^	111.7 ± 12.8^b^	< 0.001	0.62	0.28

^1^ Two-way ANOVA was used to determine the significant effects of fat amount and vitamin D content, and an interaction. ^ab^Different superscripts indicate significant difference (*P* <  0.05) by Fisher’s LSD multiple comparison test. Data are presented as means ± SEMs. ^2^White adipose tissue weight included epididymal, subcutaneous, retroperitoneum, and perinephric fats. *CON* 10% kcal fat diet, *HFD* 45% kcal fat diet; *LVd* Low vitamin D, *CVd* Control vitamin D, *HVd* High vitamin D

### Serum 25(OH)D concentration

**Fig. 1:**
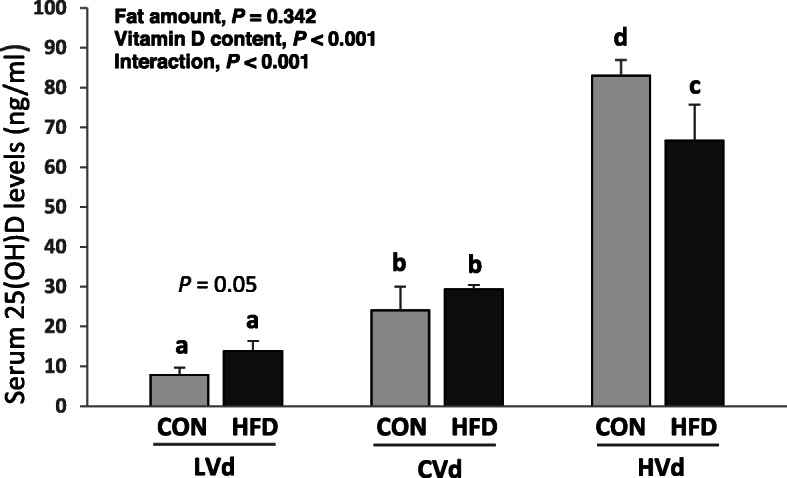
Serum 25(OH)D levels (ng/mL). Data are presented as means ± SEMs, *n* = 6 for each group. Two-way ANOVA was used to determine the significant effects of dietary fat amount and vitamin D content, and an interaction. ^ab^Different superscripts indicate significant difference (*P* < 0.05) by Fisher’s LSD multiple comparison test. CON: 10% kcal fat diet; HFD: 45% kcal fat diet; LVd: Low vitamin D; CVd: Control vitamin D; HVd: High vitamin D

Overall, dietary VD content had a significant effect (*P* <  0.001) on serum 25(OH)D levels (Fig. 1). The average values of serum 25(OH)D were 10.8 ng/mL in LVd group, 26.7 ng/mL in CVd group, and 74.9 ng/mL in HVd group. An increase in dietary VD content resulted in higher serum 25(OH)D levels in both the CON and HFD groups; however, the 25(OH)D levels showed different patterns between the two depending on the dietary VD content (interaction, *P* <  0.001). In the LVd groups, the serum 25(OH)D level tended to be higher in the HFD-LVd group compared with the CON-LVd group (*P* = 0.05, 76.6% higher). On the other hand, when the VD level in the diet was at a supplementary level, the HFD-HVd group had significantly lower serum 25(OH)D level than the CON-HVd group (*P* <  0.001, 19.7% lower), in spite of higher daily VD intake in HFD-HVd group (82.5 IU/day) compared with the CON-HVd group (71.3 IU/day). Serum 25(OH)D levels in HVd group negatively correlated with body weight (g) and white adipose tissue (g) (*r* = − 0.762, *P* <  0.01; *r* = − 0.833, *P* <  0.01, respectively). With the control level of dietary VD (in the CVd groups), there was no significant difference in the serum 25(OH)D concentration between the CON-CVd and HFD-CVd groups.

### The mRNA levels of genes involved in vitamin D absorption in small intestine

**Fig. 2:**
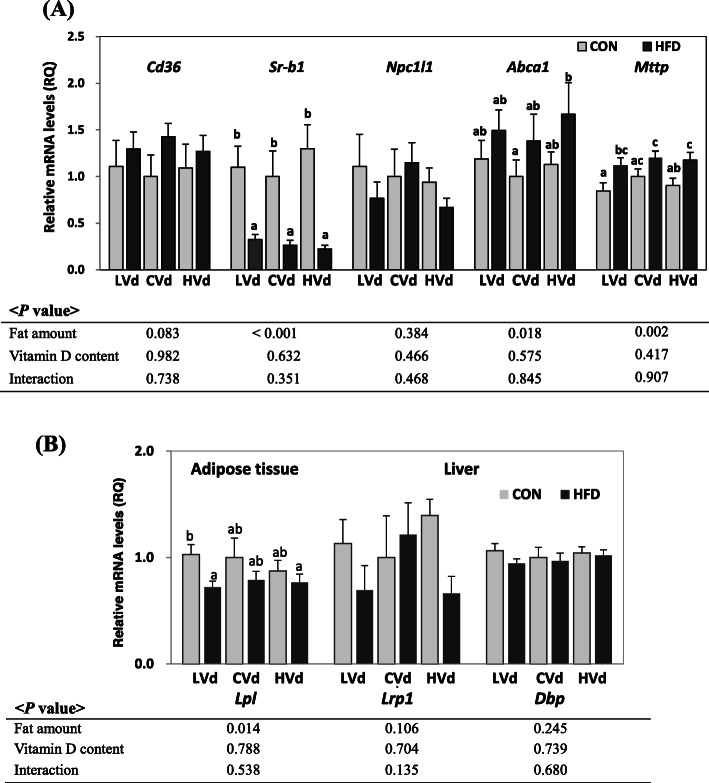
The mRNA levels of genes involved in (A) vitamin D absorption in jejunum and (B) vitamin D transport in epididymal adipose tissue and liver. Data are presented as means ± SEMs, *n* = 6 ~ 8 for each group. Two-way ANOVA was used to determine the significant effects of dietary fat amount and vitamin D content, and an interaction. ^ab^Different superscripts indicate significant difference (*P* < 0.05) by Fisher’s LSD multiple comparison test. CON: 10% kcal fat diet; HFD: 45% kcal fat diet; LVd: Low vitamin D; CVd:Control vitamin D; HVd: High vitamin D, *Cd36*, cluster of differentiation 36; *Sr-b1*, Scavenger receptor class B type1; *Npc1l1*, Niemann-Pick C1-Like 1*; Abca1*, ATP-binding cassette transporter A1; *Mttp*, Microsomal triglyceride transfer protein; *Lpl,* lipoprotein lipase; *Lrp1,* Low density lipoprotein receptor-related protein 1; *Dbp,* vitamin D binding protein

The expression levels of cholesterol transporter genes (*Cd36, Sr-b1, Npc1l1*, and *Abca1*) and *Mttp* in the jejunum were determined to investigate the effects of dietary VD content or fat amount on VD absorption in the small intestine **(**Fig. 2A). Among the cholesterol transporter genes, the mRNA levels of *Abca1* in the jejunum were significantly higher and *Cd36* mRNA levels tended to be higher (*P* = 0.08) in the HFD groups compared with the CON groups, but the mRNA levels of *Sr-b1* were significantly lower. The expression levels of *Mttp* (essential for the assembly of apolipoproteins and lipid into chylomicrons) in the jejunum were higher in the HFD groups than the CON groups (*P* = 0.002). Dietary VD content did not affect the expression of genes involved in VD absorption.

### The mRNA levels of genes involved in vitamin D transport

The expression of genes involved in the chylomicron hydrolysis and VD transport was determined. mRNA levels of epididymal *Lpl* were significantly lower in the HFD groups than the CON groups, while dietary VD content had no effect on epididymal *Lpl* expression levels. mRNA levels of liver *Lrp1* and *Dbp* were not significantly affected by either dietary fat amount or VD contents (Fig. 2B).

### Liver and adipose tissue vitamin D**3** and 25(OH)D**3** levels

#### Optimization of analytical instrument condition and method validation

For the PTAD-derivatized target compounds (VD3, d3-VD3, 25(OH)D3, and d3–25(OH)D3), a full scan analysis was conducted in which both of them were ionized with protons ([M + H]+) in ESI-positive mode. Those in ionization form were selected as the precursor ions, and a product ion scan was performed with various collision energy levels to select the quantifier ions by considering sensitivity and selectivity. MRM transitions and retention time (t_R_) for PTAD-VD3, PTAD-25(OH)D3, and their internal standards are given in an Additional file 1: Table S3. Using the matrix-matched standard, the limits of quantitation (LOQs) of VD3 and 25(OH)D3 were determined with a signal-to-noise ratio ≥ 10 for each peak in the chromatogram. The LOQs of VD3 and 25(OH)D3 in the liver and adipose tissue samples were 1 ng/mL. The linear ranges for the samples were 1~1000 ng/mL and correlation coefficients (r^2^) were determined for the linearity of the calibration curve; r^2^ for both liver and adipose tissue was ≥0.990.

The accuracy determined from the levels of three spikes (1.5, 7.5, and 75 ng/g tissue) was 96 ~ 103% and 97 ~ 107% in adipose tissue and 96 ~ 107% and 96 ~ 103% in liver tissue for VD3 and 25(OH)D3, respectively (Additional file 1: Table S4). The precision (within-day and between-day) of VD3 and 25(OH)D3 from liver and adipose tissue is shown in Table S5 in the Additional file 1.

#### Concentration and total amount of vitamin D**3** in liver and epididymal adipose tissue

VD3 concentration (ng/g tissue) and total amount (ng) in liver and epididymal adipose tissue are reported in Table 2. In the liver and adipose tissue, VD3 levels were under the LOQ in the LVd groups but were 43 and 85 times higher, respectively, in the CON-HVd group compared with the CON-CVd group and 72 and 124 times higher, respectively, in the HFD-HVd group compared to the HFD-CVd group. The dietary fat amount did not have a significant effect on VD3 level per weight in liver and adipose tissue, but its effect on the total amount in the tissue was different depending on the dietary VD content.

**Table 2:** Vitamin D3 levels and total amount in liver and epididymal adipose tissue^1,2^

		CVd		HVd		***P***-value		
		**CON** **(*n*** **= 7)**	**HFD** **(*n*** **= 7)**	**CON** **(*n*** **= 7)**	**HFD** **(*n*** **= 7)**	**Fat amount**	**Vitamin D content**	**Interaction**
**Liver**	**Vitamin D3level** **(ng/g tissue)**	14.53 ± 3.0^a^	11.09 ± 2.9^a^	628.3 ± 74.7^b^	797.0 ± 105.3^b^	0.210	< 0.001	0.192
	**Total Vitamin D3amount (ng)**	16.73 ± 3.16^a^	24.47 ± 5.70^a^	727.1 ± 108.9^b^	1608.0 ± 207.6^c^	0.001	< 0.001	0.001
**Epididymal adipose tissue**	**Vitamin D3levels** **(ng/g tissue)**	3.80 ± 0.44 ^a^	2.50 ± 0.50 ^a^	327.4 ± 61.57 ^b^	310.5 ± 28.95 ^b^	0.768	< 0.001	0.806
	**Total Vitamin D3 amount (ng)**	4.68 ± 0.54^a^	3.87 ± 0.71 ^a^	385.4 ± 45.1^b^	554.3 ± 23.3^c^	0.002	< 0.001	0.002

^1^Two-way ANOVA was used to determine the significant effects of fat amount and vitamin D content, and an interaction. ^ab^Different superscripts indicate significant difference (*P* < 0.05) by Fisher’s LSD multiple comparison test. Data are presented as means ± SEMs

^2^*CON* 10% kcal fat diet, *HFD* 45% kcal fat diet, *CVd* Control vitamin D, *HVd* High vitamin D

**Fig. 3:**
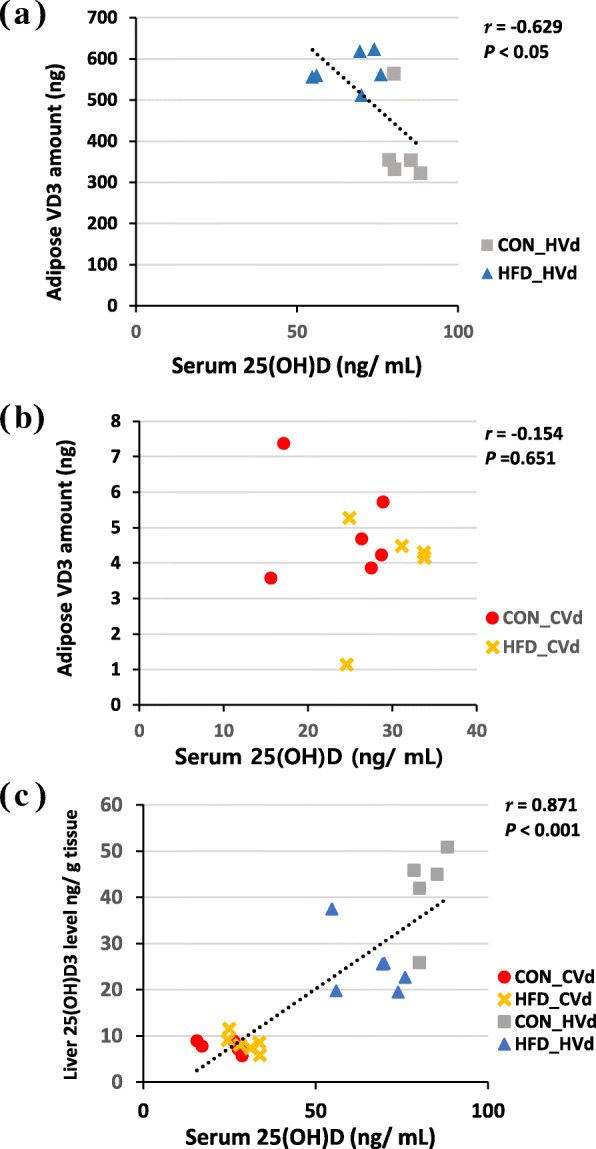
Correlations between serum 25(OH)D levels (ng/mL) and adipose tissue vitamin D3 amount in HVd groups (**a**) and CVd groups (**b**) and liver 25(OH)D3 levels (ng/g tissue)(**c**) *n* = 11 ~ 28, Pearson correlation coefficient*, r*, and *P* value are indicated

For the total VD3 amount in liver and adipose tissue, there was a significant interaction between the dietary fat amount and VD content (*P* <  0.01). In the HVd groups, the total amount of VD3 in the liver and adipose tissue were significantly higher in the HFD-HVd group (121 and 44%, respectively) compared with the CON-HVd group. The total amount of VD3(g) in adipose tissue in HVd groups showed negative correlation with serum 25(OH)D levels (ng/ mL) (*r* = − 0.629, *P* <  0.05) (Fig. 3a). However, when mice were fed the control level of VD (the CVd groups), dietary fat content did not affect the VD3 amount in liver and adipose tissue. In CVd groups, no significant correlation between the total adipose tissue VD3 amount (g) and serum 25(OH)D (ng/mL) was observed (Fig. 3b).

#### Concentration and total amount of 25(OH)D**3** in liver and epididymal adipose tissue

The 25(OH)D3 concentration (ng/g tissue) and total amount (ng) in liver and adipose tissue are summarized in Table 3. Liver and adipose tissue 25(OH)D3 concentrations in the LVd groups were < 1 ng/g tissue but were 5.6 times higher in the liver in the CON-HVd group compared to the CON-CVd group (*P* <  0.05), and 3.3 times higher in HFD-HVd group than in HFD-CVd group. There was an interaction between the dietary fat amount and VD content regarding liver 25(OH)D3 levels: the level was significantly lower in the HFD-HVd group compared with the CON-HVd group, which was not observed in the CVd groups. Liver 25(OH)D3 levels (ng/g tissue) correlated positively with serum 25(OH)D levels (ng/mL) (*r* = 0.871, *P* <  0.001) (Fig. 3c). In epididymal adipose tissue, 25(OH)D3 concentration was higher in the HVd groups compared with the CVd groups (*P* <  0.05). The dietary fat amount had no significant influence on adipose tissue 25(OH)D3 concentration.

**Table 3:** 25(OH)D3 levels and total amount in liver and epididymal adipose tissue^1,2^

		CVd		HVd		***P***-value		
		**CON** **(*n*** **= 7)**	**HFD** **(*n*** **= 7)**	**CON** **(*n*** **= 7)**	**HFD** **(*n*** **= 7)**	**Fat amount**	**Vitamin D content**	**Interaction**
**Liver**	**25(OH)D3levels** **(ng/g tissue)**	7.47 ± 0.43^a^	8.24 ± 0.67^a^	41.9 ± 3.5^c^	27.0 ± 3.0^b^	0.004	< 0.001	0.002
	**Total 25(OH)D3amount (ng)**	8.65 ± 0.88^a^	18.94 ± 1.82^a^	47.8 ± 4.3^b^	53.5 ± 6.2^b^	0.052	< 0.001	0.552
**Epididymal adipose tissue**	**25(OH)D3levels** **(ng/g tissue)**	3.43 ± 0.43 ^a^	8.03 ± 1.52 ^a^	18.1 ± 2.2 ^b^	19.5 ± 2.1 ^b^	0.089	< 0.001	0.364
	**Total 25(OH)D3amount (ng)**	4.41 ± 0.77^a^	12.5 ± 2.1^b^	22.4 ± 3.0^c^	35.9 ± 3.9^d^	0.001	< 0.001	0.334

^1^Two-way ANOVA was used to determine the significant effects of fat amount and vitamin D content, and an interaction. ^ab^Different superscripts indicate significant difference (*P* < 0.05) by Fisher’s LSD multiple comparison test. Data are presented as means ± SEMs.^2^*CON* 10% kcal fat diet, *HFD* 45% kcal fat diet, *CVd* Control vitamin D, *HVd* High vitamin D

### The mRNA levels of 25-hydroxylases in liver and epididymal adipose tissue

**Fig. 4:**
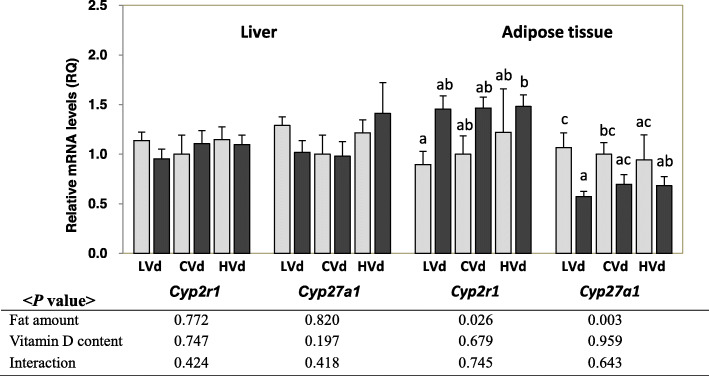
The mRNA levels of 25-hydroxylases in liver and epididymal fat tissue. Data are presented as means ± SEMs, *n* = 6 for each group. Two-way ANOVA was used to determine the significant effects of dietary fat amount and vitamin D content, and an interaction. ^ab^Different superscripts indicate significant difference (*P* < 0.05) by Fisher’s LSD multiple comparison test. CON: 10% kcal fat diet; HFD: 45% kcal fat diet; LVd: Low vitamin D group; CVd: Control vitamin D group; HVd: High vitamin D group. *Cyp2r1*, cytochrome P450 2R1; *Cyp27a1*, cytochrome P450 27A1

Neither dietary fat amount nor dietary VD content had a significant impact on liver mRNA levels of *Cyp2r1* and *Cyp27a1* (Fig. 4). On the other hand, the mRNA levels of epididymal fat *Cyp2r1* was higher (*P* = 0.026) and epididymal fat *Cyp27a1* was lower (*P* = 0.003) in the HFD groups compared with the CON groups.

## Discussion

This study showed that obesity differentially affected serum 25(OH)D levels and the total VD amount in the liver and adipose tissue depending on the levels of VD in the diet. When VD intake was at a supplementary level, high-fat diet-induced obesity resulted in a greater amount of VD3 storage in liver and epididymal adipose tissue compared with the non-obese control, and this could contribute to low serum 25(OH)D levels. However, when VD intake was at a physiological level, there was no significant difference in the serum 25(OH)D levels between the obese and the control mice.

Serum 25(OH)D has a half-life of approximately 15 days and is the best indicator for the nutritional status of VD, and our results confirm a dose-response relationship of serum 25(OH)D and dietary VD levels [23, 24]. When VD content was at 50 IU VD/kg diet (the LVd group), the serum 25(OH)D level was 59% lower than that in the CVd group (1000 IU VD/kg diet; the National Research Council’s nutrient requirement). With a supplementary level of VD (25,000 IU VD/kg diet; the HVd group), the serum 25(OH)D level was 180% higher compared with the CVd group. These results are consistent with many previous studies which have reported a positive correlation between dietary VD intake and circulating 25(OH)D levels in humans [24, 25] and rodents [26, 27].

The VD levels in liver and adipose tissue correlated positively with dietary VD content. Some previous studies have also shown that dietary VD increases tissue VD and 25(OH)D levels in a dose-dependent manner [12, 28]. VD3 levels (ng/g tissue) in the adipose tissue from the CON-HVd and HFD-HVd groups were 85 and 124 times higher than those from the CON-CVd and HFD-CVd groups, while adipose tissue 25(OH)D3 levels (ng/g tissue) were 5.3 and 2.4 times higher, respectively, in CON-HVd and HFD-HVd groups compared with the CON-CVd and HFD-CVd groups. These results indicated that supplementary VD is mostly reflected in tissue VD3 levels rather than 25(OH)D levels in serum or tissue. Lipkie et al. [12] reported that VD3 levels in rat liver and adipose tissue were 3.9 and 7.6 ng/g tissue, respectively, and 25(OH)D levels in liver and adipose tissue were less than 1 ng/g tissue when rats were fed 740 IU VD3/ kg diet for 8 wk. The VD3 concentrations in pigs fed 2000 IU VD3/day were 2.7 ng/g tissue in liver tissue and 7 ng/g tissue in adipose tissue [29]. VD and 25(OH)D levels from previous studies were similar to the levels observed in the CVd group in this study.

Depending on the dietary VD levels, the fat amount in the diet had differential effects on serum 25(OH)D levels. When dietary VD content was low (LVd groups) or at a control level, dietary fat intake did not affect serum 25(OH)D levels. However, in the HVd group, HFD-HVd group had significantly lower 25(OH)D level compared with CON-HVd group and serum 25(OH)D showed negative correlations with body weight and white adipose tissue weight. Since liver 25-hydroxylase mRNA levels were not different among the groups, the differences in serum 25(OH)D levels could be due to differences in the amount of absorbed VD, which serves as a substrate for 25(OH)D synthesis in liver. The effects of HFD-induced obesity on serum 25(OH)D levels and liver 25-hydroxylases are not well characterized and still remain contradictory. When mice were fed HFD with a control level of vitamin D, several studies reported no significant decrease of serum 25(OH)D [30–32] which is consistent with the results from this study, but decreased level of blood 25(OH)D followed by low liver *Cyp2r1* mRNA level in female obese mice was also reported [33]. Not only the expression level but also activity and genetic variants of liver *Cyp2r1* are important factors in vitamin D metabolism [34, 35], therefore, further studies focused on the role of 25-hydroxyases in obesity are needed.

In spite of 16% higher VD intake (IU/g) in HFD-HVd group compared with CON-HVd group, the serum 25(OH)D level was significantly lower (19% lower) in the HFD-HVd group. HFD-HVd group’s total VD3 amount in the liver and epididymal adipose tissue were 121 and 44% higher, respectively, compared with the CON-HVd group. Since epididymal adipose tissue accounts for less than 20% of total body adipose tissue, total body fat mass was estimated using a DEXA scan data. Average total body fat mass of the HFD-HVd group was 11.6 g and that of the CON-HVd group was 5.2 g [See additional file 1: Table S6]. By using the epididymal fat VD3 level (ng/g tissue) and total body fat mass, the estimated total adipose tissue VD3 amount was calculated and it was 192% higher in the HFD-HVd group than that in the CON-HVd group (8187 vs. 2799 ng, respectively). These results suggested that obesity could lead to a larger amount of VD stored in the liver and adipose tissue, which could contribute to a low serum 25(OH)D level when VD intake was at a supplementary level. However, only two animals per group were scanned with DEXA, therefore total adipose tissue VD amount based on DEXA is a rough estimation. The limitation of this study is that subcutaneous fat VD content was not measured. There are mixed results regarding the VD content in the subcutaneous and visceral fat. Carrelli et al. [13] reported that VD concentration was higher in omental fat compared with subcutaneous fat in human subjects, while Lawson et al. [36] found that VD in perirenal fat (visceral fat) was lower than in axillary fat (subcutaneous fat). Therefore, further studies are needed for the accurate estimation of total adipose tissue VD amount. Nevertheless, results from this study shows that more VD is stored in the liver and epididymal adipose tissue due to fatness.

Wortsman et al. [10] also reported that the serum 25(OH)D level was lower in obese persons than non-obese persons after a supplementary high dose of VD (50,000 IU); they assumed that this could be due to VD sequestration in the adipose tissue, however, VD in the tissue was not measured in their study. In this study, by quantifying VD from liver and adipose tissue, it was confirmed that increased tissue VD amount in obesity could contribute to a lower the serum 25(OH)D level when VD was at a supplementary level. However, contrary to the HVd groups, there was no significant difference in the liver and adipose tissue VD amount between the CON-CVd and HFD-CVd groups. According to Heaney et al. [37], dietary VD is slowly converted to circulating 25(OH)D and VD seemed to be stored in adipose tissue when VD input is high whereas there is rapid conversion of VD to 25(OH)D when VD input is at a physiologic level (a daily input of 2000 IU). Therefore, the results from this study and others suggest that serum 25(OH)D could be lower in the obese due to VD distribution into a larger mass of adipose tissue rather than sequestration, and the rate at which VD is distributed to the blood and adipose tissue could be dependent on dietary VD content.

Contrary to the VD-supplemented groups, animals fed the deficient level of VD (50 IU/kg diet) with HFD tended to have a higher serum 25(OH)D level compared with the CON-LVd group (*P* = 0.054). The tissue VD levels of the VD-deficient groups could not be determined because of the low levels of VD (under LOQ < 1 ng/g tissue) present in the tissue. Another limitation of this study is that the association between serum 25(OH)D and tissue VD levels could not be determined when dietary VD was at a deficient level. VD is preferentially stored in the blood and rapidly converted to 25(OH)D with low VD intake rather than being stored in the body fat [37]. A previous study has also reported that tissue VD or 25(OH)D levels in rats fed 49 IU ergocalciferol (VD2) for 8 weeks were lower than 2 or 0.5 ng/g tissue, respectively [12]. Therefore, it is possible that most of the absorbed dietary VD was converted to serum 25(OH)D in mice fed a 50 IU VD/kg diet.

Serum 25(OH)D level of HFD-LVd group was 78.2% higher than that of CON-LVd group, although the amount of daily VD intake was 17% higher in the HFD-LVd group compared to the CON-LVd group. Therefore, tendency of higher serum 25(OH)D level in HFD-LVd group than CON-LVd group could be ascribed to various factors such as adiposity or the fat content of a meal as well as the amount of dietary VD intake. There are two possible mechanisms which could have contributed to this higher VD absorption in HFD-LVd group. First, high fat content in the diet could have increased the absorption of VD. Dietary fat has been reported to stimulate bile acid secretion and consequently enhance VD absorption [38]. Higher serum 25(OH)D levels was observed when people took VD with a high-fat meal [18] and it was reported that more VD was absorbed when taken with fat-containing meal than fat-free meal [39]. However, more studies are needed to determine the influence of dietary fat on VD absorption because the effect of fat on VD absorption was not identified in some study [40] and correlation between dietary fat amount and VD absorption was not found [19]. Second, the jejunal mRNA levels of the genes involved in VD absorption and chylomicron formation in small intestine were higher in the HFD groups compared with the CON groups. MTP is essential for chylomicron formation and it increases fat absorption at small intestine [15, 41], and mRNA level of *Mttp* was significantly higher in HFD group than in CON group, especially HFD-LVd group showed 32% higher levels of *Mttp* compared with the CON-LVd group. This result is consistent with previous study in which increased *Mttp* mRNA and protein levels in jejunum of HFD-induced obese animal were observed [42–45], therefore it is suggested that HFD-induced obesity could have contributed to the increase in VD absorption by increasing fat absorption. In addition, it has been recently reported that VD is absorbed into the intestine not only by passive diffusion but also by cholesterol transporter proteins [20, 21]. In this study, mRNA levels of *Abca1* were higher, *Cd36* tended to be higher, and *Sr-b1* were lower in HFD groups compared with CON groups. There is a possibility that obesity could have affected VD absorption through cholesterol transporters. Higher *Cd36* mRNA levels been reported in obese mice [45]. It is hard to figure out the effect of changes in *Sr-b1* on VD absorption, because SR-B1 has been reported to be involved in both influx and efflux of VD at the intestinal apical membrane [20]. Therefore, further studies including protein expression are needed to elucidate the role of each cholesterol transporter in VD transport.

## Conclusion

At supplementary level of VD intake, high fat diet-induced obesity resulted in lower serum 25(OH)D levels than the lean control. A significant amount of dietary VD is stored in the liver and adipose tissues when VD is at a supplementary level; thus excess body adiposity could contribute to lower serum 25(OH)D level. Whereas, when VD in the diet is at a control (NRC requirement, 1000 IU/kg diet) level, there was no significant effect of obesity on tissue VD amount and serum 25(OH)D levels. These finding have expanded our knowledge that dietary VD distribution into the blood and adipose shows differential response to dietary vitamin D intake depending on the adiposity. In addition, there is a possibility that obese subjects will have to increase their VD intake when supplementing VD, if they expect the same effect as non-obese subjects.

## Supplementary information

**Additional file 1.**

## Data Availability

The datasets during and/or analyzed during the current study available from the corresponding author on reasonable request.

## Abbreviations

25(OH)D: 25-hydroxyvitamin D
25(OH)D3: 25-hydroxycholecalciferol
Abca1: ATP-binding cassette transporter
AcN: Acetonitrile
CD36: Cluster of differentiation 36
CON: Control diet
CV: Coefficient of variation
CVd: Control vitamin D
Cyp2r1: Cytochrome P450 2R1
Cyp27a1: Cytochrome P450 27A1
Dbp: Vitamin D binding protein
HFD: High-fat diet
HVd: High vitamin D
LC/MS: Liquid chromatography/mass spectrometry
LPL: Lipoprotein lipase
LOQ: Limit of quantitation
Lrp1: Low density lipoprotein receptor-related protein 1
LVd: Low vitamin D
MRM: Multiple reaction monitoring
MTBE: Methyl tert-butyl ether
MTP: Microsomal triglyceride transfer protein
NPC1L1: Niemann-Pick C1-Like 1
QC: Quality control
RIA: Radioimmunoassay
SR-B1: Scavenger receptor class B type 1
UHPLC: Ultra-high-performance liquid chromatography
UVB: Ultraviolet B
VD: Vitamin D
VD2: Ergocalciferol
VD3: Cholecalciferol
