# Supplementary material for: Effects of high fat diet-induced obesity on vitamin D metabolism and tissue distribution in vitamin D deficient or supplemented mice
Source: Nutr Metab (Lond). 2020 Jun 15;17:44. doi: 10.1186/s12986-020-00463-x (PMC7294642; doi:10.1186/s12986-020-00463-x)
Supplement: Supplementary file 1 — Additional file 1. [file 12986_2020_463_MOESM1_ESM.docx]

**Additional file 1**

**Method**

**Sample preparation and PTAD derivatization**

The QC samples were homogenized (IKA homogenizer, Sigma-Aldrich) in PBS to 33% (w/w) solutions, and the homogenates were stored at -80 °C until analyzed. For the quantification of VD3 in epididymal adipose tissue or liver tissue, tissue samples were homogenized in PBS to 33% (w/w) solutions. The weight of the tissue was recorded previously to determine the mass fraction of the tissue in the homogenate. 400 μL of homogenate from adipose tissue or liver sample was spiked with 10 μL of internal standard containing 1 μg/ mL d3-VD3 and d3-25(OH)D3 in AcN, mixed, then incubated for 15 min. 400 μL of AcN was added, after which the mixture was vortexed for 5 min and centrifuged for 1 min (6,000 x g, 4°C). MTBE (400 μL) was added, then the homogenate was vortexed for 5 min and centrifuged for 1 min (6,000 x g, 4 °C). The upper layer was collected and further extraction from the bottom layer was repeated twice using AcN, MTBE, and PBS. The solvent in the upper layer was evaporated off with N_2_ gas and the residue reconstituted with methanol (700 μL) and K_2_HPO_4_ (0.4 M, 300 μL). Solid-phase extraction was performed on Oasis HLB cartridges (1 cc, 3 mL, Waters, Milford, MA, USA) via a vacuum manifold (Supleco, Park Bellefonte, PA, USA). Cartridges were conditioned by sequential washing with 1 mL each of ethanol, methanol, and water, then loaded with 1 mL of reconstituted sample, washed with 1 mL methanol (30%) and an additional 1 mL of methanol (liver: 70%, adipose tissue: 60%), and eluted with AcN (liver: 2 mL, adipose tissue: 3 mL). The solvent in the eluted samples was evaporated off with N_2_ gas and the sample reconstituted with 100 μL of 4 mg PTAD/mL dry-AcN. For PTAD derivatization, samples were vortexed for 1 h and stored at -80 °C until analyzed.

**Table S1. Composition of experimental diets^1^**

|  | CON (10% kcal fat) | | | HFD (45% kcal fat) | | |  |
| --- | --- | --- | --- | --- | --- | --- | --- |
|  | | LVd  (50 IU/kg diet) | CVd  (1,000 IU/kg diet) | HVd  (25,000 IU/kg diet) | LVd  (50 IU/kg diet) | CVd  (1,000 IU/kg diet) | HVd  (25,000 IU/kg diet) |
| Casein (g) | 200 | 200 | 200 | 200 | 200 | 200 |  |
| L-Cystine (g) | 3 | 3 | 3 | 3 | 3 | 3 |  |
| Sucrose (g) | 350 | 350 | 350 | 172.8 | 172.8 | 172.8 |  |
| Cornstarch (g) | 315 | 315 | 315 | 72.8 | 72.8 | 72.8 |  |
| Dyetrose (g) | 35 | 35 | 35 | 100 | 100 | 100 |  |
| Soybean Oil (g) | 45 | 45 | 45 | 45 | 45 | 45 |  |
| t-BHQ (g) | 0.009 | 0.009 | 0.009 | 0.009 | 0.009 | 0.009 |  |
| Lard (g) | - | - | - | 157.5 | 157.5 | 157.5 |  |
| Cellulose (g) | 50 | 50 | 50 | 50 | 50 | 50 |  |
| Mineral Mix (g)^2^ | 35 | 35 | 35 | 35 | 35 | 35 |  |
| Vitamin Mix (g)  (No VD) | 10 | - | 10 | 10 | - | 10 |  |
| Vitamin Mix^3^ (g) | - | 10 | - | - | 10 | - |  |
| VD3 Premix (100 IU/g) | 0.5 | - | - | 0.5 | - | - |  |
| VD3 (400,000 IU/g) | - | - | 0.0625 | - | - | 0.0625 |  |
| Choline Bitartrate (g) | 2 | 2 | 2 | 2 | 2 | 2 |  |
| Total (g) | 1,045.5 | 1,045 | 1,045 | 848.6 | 848.1 | 848.2 |  |
| Protein (% kcal) | 20.0 | 20.0 | 20.0 | 20.2 | 20.2 | 20.2 |  |
| Fat (% kcal) | 10.0 | 10.0 | 10.0 | 45.4 | 45.4 | 45.4 |  |
| Carbohydrate (% kcal) | 70.0 | 70.0 | 70.0 | 34.4 | 34.4 | 34.4 |  |
| kcal/g diet | 3.69 | 3.69 | 3.69 | 4.64 | 4.64 | 4.64 |  |

^1^ Resource: Dyets, Inc., Bethlehem, PA, USA ^2.^35 g of mineral mix (Dyets, #200000) provides 5.2 g calcium, 4 g phosphorus, 3.6 g potassium, 1 g sodium, 1.6 g chloride, 0.3 g sulfur, 0.5 g magnesium, 35 mg iron, 6 mg copper, 54 mg manganese, 30 mg zinc, 2 mg chromium, 0.2 mg iodine, 0.1 mg selenium, and 4.2 g sucrose. ^3^10 g of vitamin mix (Dyets, #300050) provides 4,000 IU vitamin A, 1,000 IU vitamin D3, 50 IU vitamin E, 30 mg niacin, 16 mg pantothenic acid, 7 mg vitamin B6, 6 mg vitamin B1, 6 mg vitamin B2, 2 mg folic acid, 0.8 mg menadione, 0.2 mg biotin, 10 μg vitamin B12, and 9.8 g sucrose. (VD3: vitamin D3).

**Table S2. Primer sequences used in real-time PCR^1^**

| Gene ^1^ | Forward primer | Reverse primer |
| --- | --- | --- |
| *Cyp2r1* | TGGTGAGGTAAATGAGGCTTTC | TGCCAGTGCTCCAGTCTTC |
| *Cyp27a1* | CCAAGGCAAGGTGGTAGAGA | CTTCATCGCACAAGGAGAGC |
| *Mttp* | TCTGGCTGAGGTGGGAATAC | CACTCAGGCAATTCGAGACA |
| *Sr-b1* | AATGCTCCTTTGGGTTAGGG | GCCCCCGATACTCTGTTTG |
| *Npc1l1* | TGTTTGGTATGGAGAGTGTGGA | GTCACAGCAGAGACTGACATTG |
| *Cd36* | CCAAGCTATTGCGACATGATT | TCTCAATGTCCGAGACTTTTCA |
| *Abca1* | CCTCACATCCTCATCCTCGT | CGCTCTCTTGGGACTTGGTA |
| *Lpl* | GGGGTTTTCTTCATTCAGCA | ACACATTTACCAGGGGGTCA |
| *Lrp1* | GACCAGGTGTTGGACACAGATG | AGTCGTTGTCTCCGTCACACTTC |
| *Dbp* | GTCCGAGTGTTTCTCCACCA | TCTGTTGCCTGTTTCAGCAC |
| *Gapdh* | GGAGAAACCTGCCAAGTA | AAGAGTGGGAGTTGCTGTTG |

^1^ *Cyp2r1*, cytochrome P450 2R1; *Cyp27a1*, cytochrome P450 27A1; *Mttp*, Microsomal triglyceride transfer protein; *Sr-b1*, Scavenger receptor class B type1 ; *Npc1l1*, Niemann-Pick C1-Like 1; *Cd36*, cluster of differentiation 36 ; *Abca1*, ATP-binding cassette transporter A1 ; *Lpl,* lipoprotein lipase; *Lrp1*, Low density lipoprotein receptor-related protein 1 ; *Dbp*, vitamin D binding protein; *Gapdh*, glyceraldehyde 3-phosphate dehydrogenase.

**Table S3. MRM transition^1^**

| Name | t_R_ (min) | **Mono isotopic mass** | **MRM transition**  precursor ion > product ion |
| --- | --- | --- | --- |
| Vitamin D**3**-PTAD | 14.05 | 591.4 | 591.4 > 298.1 |
| d3-Vitamin D3-PTAD | 13.99 | 594.4 | 594.3 > 301.1 |
| 25-hydroxyvitamin D3-PTAD | 7.48 | 607.4 | 607.4 > 298.1 |
| d3-25-hydroxyvitamin D3-PTAD | 7.44 | 610.4 | 610.4 > 301.1 |

^1^R.T., retention time; MRM, multiple reaction monitoring.

**Table S4. Accuracy of vitamin D3 and 25-hydroxyvitamin D3 measurements in liver and adipose tissue^1^**

|  | Matrix | Spiked level ^2^  ng/ g tissue (or ng/ mL) | Accuracy (%) |
| --- | --- | --- | --- |
| Vitamin D3 | Liver | 1.5 (2) | 106.42 |
|  |  | 7.5 (10) | 96.42 |
|  |  | 75 (100) | 103.26 |
|  | Adipose tissue | 1.5 (2) | 96.8 |
|  |  | 7.5 (10) | 102.03 |
|  |  | 75 (100) | 94.84 |
| 25-hydroxyvitamin D3 | Liver | 1.5 (2) | 103.6 |
|  |  | 7.5 (10) | 87.75 |
|  |  | 75 (100) | 99.86 |
|  | Adipose tissue | 1.5 (2) | 106 |
|  |  | 7.5 (10) | 106.34 |
|  |  | 75 (100) | 97.25 |

^1^Accuracy was repeated on three days by the following equation: (mean of measured value/ QC sample value) x 100%.

^2^Spike levels were shown both as [ng/g tissue] when extracting 400 mg of a 33% w/w tissue homogenate and [ng/ mL] for final concentration of vitamin D3 and 25(OH)D3 standards in reconstituted AcN.

**Table S5. Within day (n=3) and between day (n=3) precision of vitamin D3 and 25-hydroxyvitamin D3 measurements from liver and adipose tissue ^1^**

|  | Matrix | Spiked level ^2^  ng/ g tissue  (or ng/ mL) | Within day precision  (n =3, %CV) | Between day precision  (n =3, %CV) |
| --- | --- | --- | --- | --- |
| Vitamin D3 | Liver | 1.5 (2) | 3.78 | 15.17 |
|  |  | 7.5 (10) | 2.12 | 1.63 |
|  |  | 75 (100) | 0.31 | 6.28 |
|  | Adipose tissue | 1.5 (2) | 14.64 | 7.40 |
|  |  | 7.5 (10) | 7.37 | 6.46 |
|  |  | 75 (100) | 2.04 | 2.23 |
| 25-hydroxyvitamin D3 | Liver | 1.5 (2) | 5.91 | 4.39 |
|  |  | 7.5 (10) | 3.28 | 6.19 |
|  |  | 75 (100) | 2.77 | 8.27 |
|  | Adipose tissue | 15 (10) | 4.32 | 4.29 |
|  |  | 75 (50) | 8.22 | 0.91 |
|  |  | 375 (250) | 5.17 | 1.76 |

^1^Precision was determined as relative coefficient of variation (%CV): (standard deviation / mean) × 100%. The analyses were done on three different days

^2^ Spike levels were shown both as [ng/g tissue] when extracting 400mg of a 33% w/w tissue homogenate and [ng/ mL] for final concentration of vitamin D3 and 25(OH)D3 standards in reconstituted AcN.

**Table S6. Fat mass and lean mass measurement with DEXA scan^1^**

|  | **CON-LVd** | **CON-CVd** | **CON-HVd** |  | **HFD-LVd** | **HFD-CVd** | | **HFD-HVd** | | |
| --- | --- | --- | --- | --- | --- | --- | --- | --- | --- | --- |
| **Fat mass (g)**  1  2 | 6.6  8.3 | 6.9  6.1 | 3.9  6.4 |  | 10.7  19.2 | | 12.5  12.0 | | 12.8  10.4 |  |
| **Average** | 7.5 | 6.5 | 5.2 |  | 14.95 | | 12.3 | | 11.6 |  |
| **Lean mass (g)**  1  2 | 21.2  23.4 | 20.6  20.1 | 19.4  24 |  | 26.7  23.7 | | 27.7  26.9 | | 23.9  24.9 |  |
| **Average** | 22.3 | 20.4 | 21.7 |  | 25.2 | | 27.3 | | 24.4 |  |

^1^ Two of each group were scanned with DEXA after anesthesia with 1.5g/ kg urethane (intraperitoneal). DEXA: dual-energy X-ray absorptiometry.
